# Systematic Dissection and Trajectory-Scanning Mutagenesis of the Molecular Interface That Ensures Specificity of Two-Component Signaling Pathways

**DOI:** 10.1371/journal.pgen.1001220

**Published:** 2010-11-24

**Authors:** Emily J. Capra, Barrett S. Perchuk, Emma A. Lubin, Orr Ashenberg, Jeffrey M. Skerker, Michael T. Laub

**Affiliations:** 1 Department of Biology, Massachusetts Institute of Technology, Cambridge, Massachusetts, United States of America; 2 Howard Hughes Medical Institute, Massachusetts Institute of Technology, Cambridge, Massachusetts, United States of America; 3 Department of Bioengineering, University of California Berkeley, Berkeley, California, United States of America; 4 Physical Biosciences Division, Lawrence Berkeley National Laboratory, Berkeley, California, United States of America; University of Chicago, United States of America

## Abstract

Two-component signal transduction systems enable bacteria to sense and respond to a wide range of environmental stimuli. Sensor histidine kinases transmit signals to their cognate response regulators via phosphorylation. The faithful transmission of information through two-component pathways and the avoidance of unwanted cross-talk require exquisite specificity of histidine kinase-response regulator interactions to ensure that cells mount the appropriate response to external signals. To identify putative specificity-determining residues, we have analyzed amino acid coevolution in two-component proteins and identified a set of residues that can be used to rationally rewire a model signaling pathway, EnvZ-OmpR. To explore how a relatively small set of residues can dictate partner selectivity, we combined alanine-scanning mutagenesis with an approach we call trajectory-scanning mutagenesis, in which all mutational intermediates between the specificity residues of EnvZ and another kinase, RstB, were systematically examined for phosphotransfer specificity. The same approach was used for the response regulators OmpR and RstA. Collectively, the results begin to reveal the molecular mechanism by which a small set of amino acids enables an individual kinase to discriminate amongst a large set of highly-related response regulators and vice versa. Our results also suggest that the mutational trajectories taken by two-component signaling proteins following gene or pathway duplication may be constrained and subject to differential selective pressures. Only some trajectories allow both the maintenance of phosphotransfer and the avoidance of unwanted cross-talk.

## Author Summary

Maintaining the specificity of signal transduction pathways is critical to the ability of cells to process information, make decisions, and regulate their behavior. Preventing cross-talk often relies predominantly on molecular recognition and a set of specificity-determining residues in cognate proteins. Identifying these residues and understanding how they dictate specificity is still a major challenge. Additionally, we have a rudimentary understanding of how specificity evolves, particularly after gene duplication events. We tackled these questions using two-component signaling proteins, the largest family of bacterial signaling proteins. Using analyses of amino acid coevolution, we pinpointed a set of specificity residues in histidine kinases and their cognate substrates. Then, using systematic mutagenesis, we characterized the complete set of intermediates between two different signaling systems, EnvZ/OmpR and RstA/RstB. The results demonstrate that specificity residues contribute unequally and, importantly, that some residues depend substantially on the identity of neighboring residues. We also demonstrate how the specificity of EnvZ/OmpR can be reprogrammed to match that of RstB/RstA through a series of individual substitutions without disrupting the kinase/regulator interaction. Notably, this property is not shared by all trajectories from EnvZ/OmpR to RstA/RstB, suggesting that the duplication/divergence process that likely produced these two pathways may have been fundamentally constrained.

## Introduction

Protein-protein interactions are crucial to virtually every cellular process. Within the crowded confines of the cell, proteins must distinguish between their cognate partners and non-cognate partners, in order to avoid unproductive and potentially deleterious interactions. The problem of interaction specificity is particularly acute for paralogous protein families where proteins with diverse cellular functions share significant structural and sequence similarity. Cells have evolved many mechanisms to cope with potential cross-talk and to ensure the specificity of protein-protein interactions [1]–[2]. In multicellular organisms, spatial mechanisms that prevent related, but distinct, proteins from coming in contact with one another are often used to create specificity. For example, scaffold proteins, the localization of proteins to different subcellular compartments, and tissue-specific expression can all insulate distinct pathways. Temporal mechanisms, such as the differential timing of expression, are also used to insulate pathways. Although cells employ each of these strategies, in many cases the primary means of preventing unwanted interactions is molecular recognition. However, our understanding of precisely how proteins discriminate between cognate and non-cognate partners at the molecular level is surprisingly rudimentary. Identifying the amino acids responsible, elucidating the precise roles played by each residue, and understanding their complex interdependencies remain major challenges for most protein-protein interactions.

Two component signal transduction pathways provide a tractable system for addressing these questions. These signaling pathways, which are the dominant form of signaling in bacteria, typically consist of a sensor histidine kinase (HK) and a cognate response regulator (RR) [3]. Upon activation of the pathway, a histidine kinase dimer will autophosphorylate on a conserved histidine that then serves as the phosphodonor for a cognate response regulator. Phosphorylation of the response regulator typically activates an output domain which can effect changes in cellular physiology, often by modulating gene expression [4]. Many histidine kinases are bifunctional and when not active for autophosphorylation, will drive the dephosphorylation of their cognate response regulators.

Two-component signaling systems are used for sensing and adapting to a wide range of environmental and intracellular stimuli [3] and most bacterial species encode dozens, if not hundreds of kinase-regulator pairs. Most histidine kinases have only one or two cognate response regulators, and there is minimal cross-talk between different pathways at the level of phosphotransfer [5], [6]. The specificity of phosphotransfer is dictated, on a system-wide level, at the level of molecular recognition [6]. That is, histidine kinases exhibit a large kinetic preference *in vitro* for their *in vivo* cognate regulator(s) relative to all other response regulators [6]–[8]. Hence, cellular context is not essential and the basis of *in vivo* phosphotransfer specificity can be dissected *in vitro*.

To identify the amino acids that govern the specificity of phosphotransfer in two-component pathways, several groups have examined patterns of amino acid coevolution in cognate pairs of histidine kinases and response regulators [9]–[12]. The rationale behind this approach is that if a residue critical to molecular recognition mutates, it must either revert or be compensated for by a mutation in the cognate protein. Many of the residues identified in these computational approaches are at the molecular interface formed in a co-crystal structure of a histidine kinase-response regulator complex [13]. However, residues in direct contact do not necessarily dictate specificity [9] and computational approaches alone cannot reveal how a histidine kinase discriminates between cognate and non-cognate substrates.

Using the *E. coli* histidine kinase EnvZ as a model, we mapped a subset of coevolving residues that are critical to the specificity of phosphotransfer [9]. Mutating as few as three residues within the DHp (Dimerization and Histidine phosphotransfer) domain of EnvZ was sufficient to reprogram its phosphotransfer specificity from OmpR to the non-cognate substrate RstA. Although a set of residues that could switch the phosphotransfer specificity of EnvZ was identified, several fundamental questions remain unanswered. Can phosphotransfer specificity also be rewired by making mutations in a response regulator? Do individual specificity residues function as positive elements to promote cognate interactions, as negative elements to prevent non-cognate interactions, or both? Do individual residues contribute equally and independently or are there “hot spots” and dependencies at the amino acid level?

Here, we couple analysis of amino acid coevolution with alanine-scanning mutagenesis and an approach we call trajectory-scanning mutagenesis to systematically dissect the basis of phosphotransfer specificity in two-component signaling pathways. The results provide new insights into how histidine kinases use a set of amino acids to “choose” their cognate substrates, and *vice versa*. The results have important implications for understanding the evolution of two-component signaling pathways and the mechanisms that cells can use to insulate pathways following gene duplication.

## Results

### Identification of coevolving residues in cognate kinase-regulator pairs

To identify the amino acids responsible for determining the specificity of phosphotransfer in two-component signaling pathways, we searched for residues that covary in cognate HK-RR pairs. Histidine kinases and response regulators that are encoded in the same operon typically form exclusive one-to-one pairings, exhibiting a highly specific interaction both *in vivo* and *in vitro*. We identified ∼4500 operonic pairs of histidine kinases and response regulators from a phylogenetically diverse set of 400 sequenced bacterial genomes. To identify coevolving residues, we concatenated cognate HK-RR pairs, performed a large multiple sequence alignment, and then measured mutual information between columns of the sequence alignment. We noted that some columns tended to have high mutual information scores with many other columns in the alignment, an observation also made in other analyses of mutual information [14]. For example, positions 8 and 270 have relatively broad score distributions with long tails, while positions 18 and 202 have narrower distributions centered closer to the origin (Figure S1A and S1B). Consequently, the pairs 8–270 and 18–202, which possess identical mutual information scores of 0.35, cannot be treated identically. We used a relatively simple correction in which raw MI scores were normalized by each column's average raw MI score with all 310 positions in the sequence alignment (Figure S1C).

**Figure 1:**
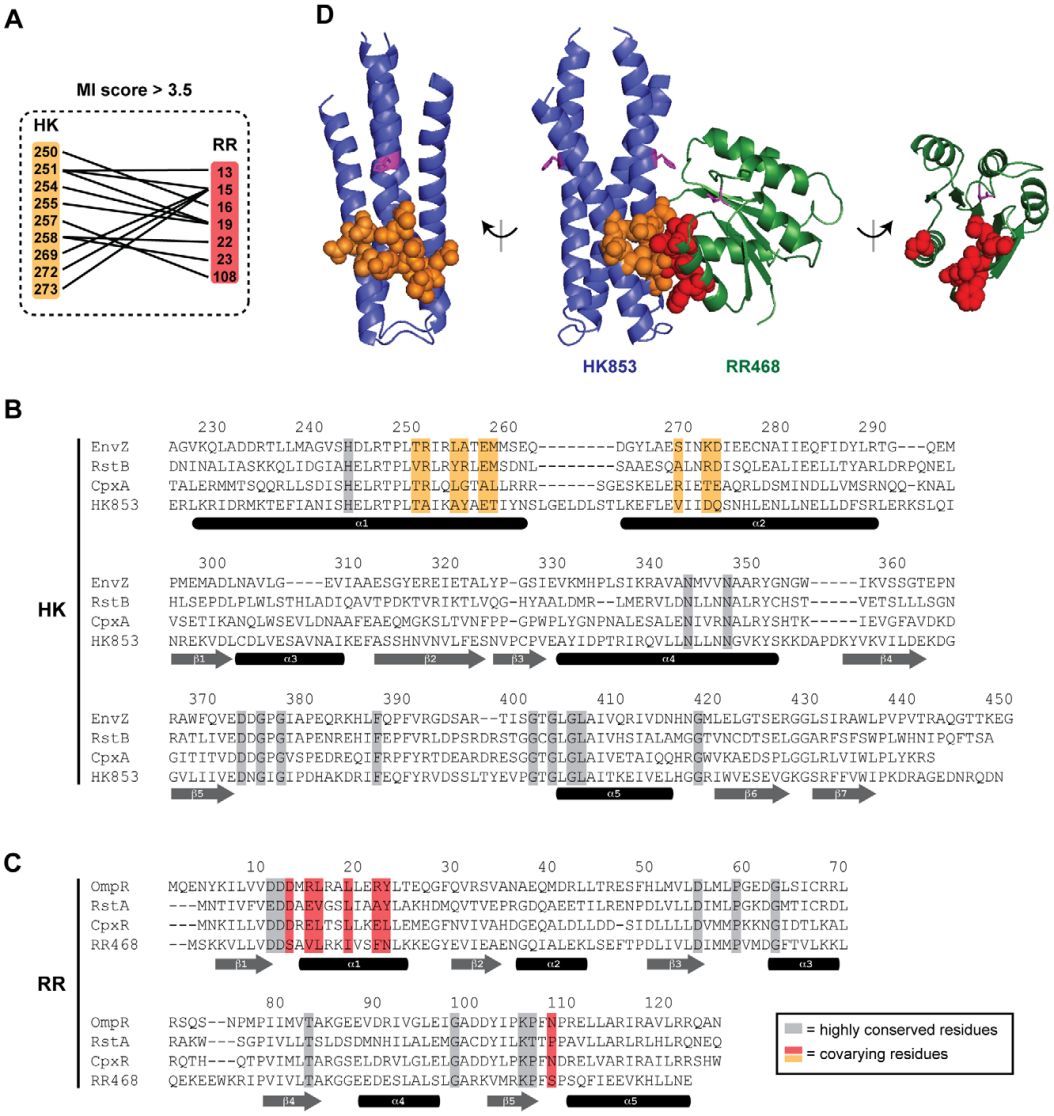
Identification of coevolving amino acids in cognate pairs of histidine kinases and response regulators. (A) Residues in histidine kinases and response regulators that strongly coevolve (adjusted MI score >3.5) are listed with lines connecting covarying pairs. Residues are numbered according to their position in *E. coli* EnvZ and OmpR. (B–C) Residues in histidine kinases that coevolve with residues in response regulators are shown on a primary sequence alignment of HK853 from *T. maritima* and EnvZ, RstB, and CpxA from *E. coli*. Residues in response regulators that strongly coevolve with residues in histidine kinases are shown on a primary sequence alignment of RR468 from *T. maritima* and OmpR, RstA, and CpxR from *E. coli*. Residues highly conserved across all two-component signaling proteins are shaded in grey. Coevolving residues are shown in orange and red for the kinase and regulator, respectively. Secondary structure elements, based on the co-crystal structure of HK853 and RR468 from *T. maritima* [13], are shown beneath the sequences. (D) Coevolving residues mapped onto the HK853-RR468 structure. Coevolving residues are shown by space-filling and colored as in panels A–C. The side chains of the conserved phosphorylatable histidines and aspartate are shown as magenta sticks. The HK853-RR468 complex is shown in the center with each individual molecule rotated 90° and shown separately.

At an adjusted score threshold of 3.5, we found 12 coevolving pairs, comprising 9 residues in the histidine kinases and 7 in the response regulators (Figure 1A–1C). These residues form a single, densely-interconnected cluster of coevolving residues. The residues are all solvent-exposed in the individual molecules, but buried within the molecular interface formed in a co-crystal structure of *T. maritima* HK853 and RR468 (Figure 1D) [13]. The residues identified here overlap substantially with, but are not identical to, those we identified previously [9]. Of the coevolving residues in the kinase, all are in the DHp domain, consistent with this domain being the primary site of interaction with the response regulator. Within the DHp domain, the coevolving residues are found on both alpha helices and are located below the histidine phosphorylation site (Figure 1D). The covarying residues in the response regulator are spatially near the conserved aspartic acid phosphorylation site (Figure 1D), predominantly on a single face of alpha helix-1 in the receiver domain with one additional residue within the β5-α5 loop. At lower score thresholds, an additional cluster of coevolving residues are found (Figure S2), but we focus here on the set of 16 residues identified at a threshold of 3.5.

### Rewiring response regulator specificity

**Figure 2:**
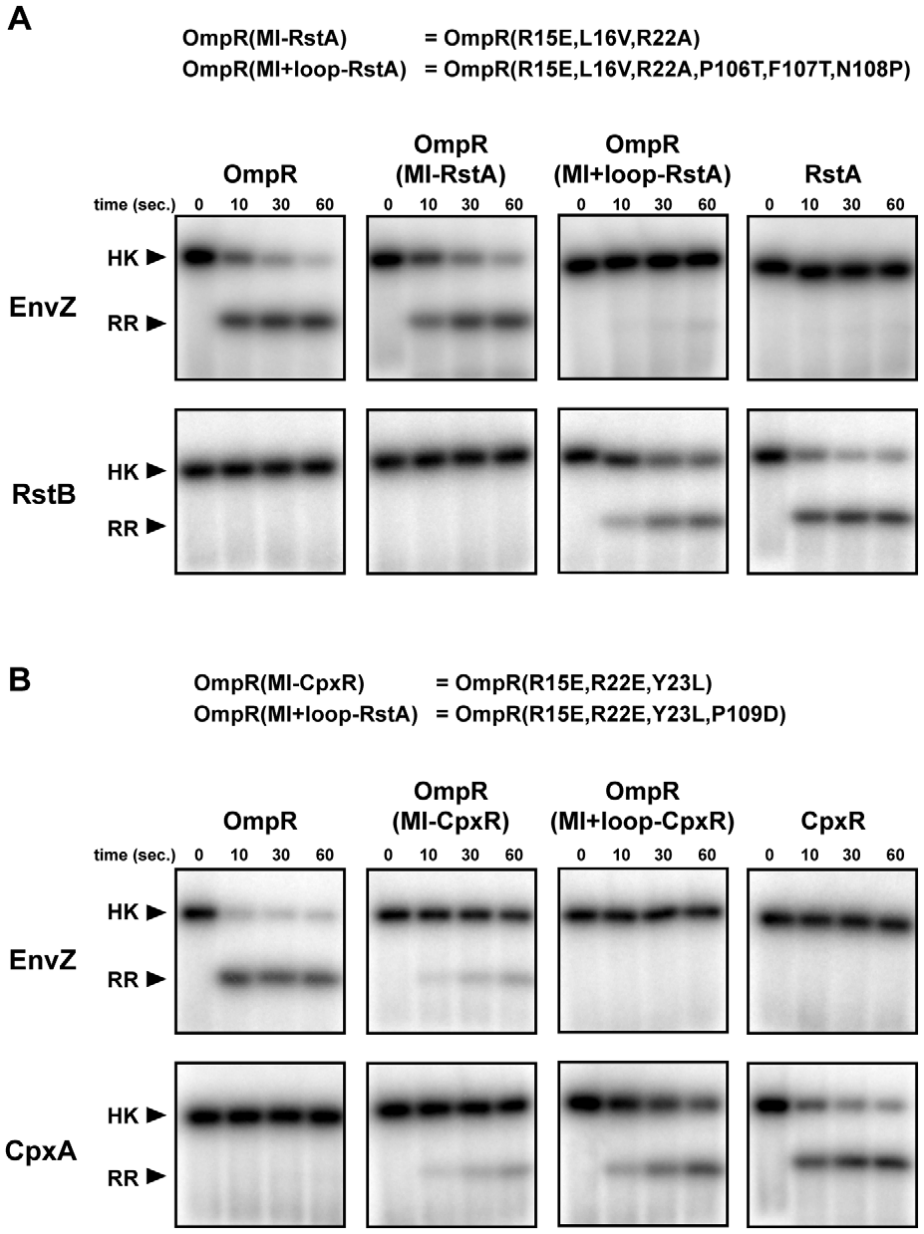
Rewiring the specificity of response regulators. (A) The histidine kinases EnvZ and RstB were autophosphorylated and examined for phosphotransfer to the response regulators indicated. The mutations in OmpR(MI-RstA) and OmpR(MI+loop-RstA) are listed at the top. (B) The histidine kinases EnvZ and CpxA were autophosphorylated and examined for phosphotransfer to the response regulators indicated. The mutations in OmpR(MI-CpxR) and OmpR(MI+loop-CpxR) are listed at the top. Each gel image shows phosphotransfer after 0, 10, 30, and 60 seconds. Bands corresponding to autophosphorylated kinases are labeled on the left. If phosphotransfer occurred, bands corresponding to the phosphorylated regulator appear below the kinase band.

Our previous studies demonstrated that many of the coevolving residues in the kinase (Figure 1) are critical to the phosphotransfer specificity of EnvZ and when mutated can reprogram its substrate selectivity [9]. To test whether we could also rewire the specificity of a response regulator, we again coupled our analyses of coevolution with site-directed mutagenesis. We aimed to mutate the response regulator OmpR such that it was no longer phosphorylated by its cognate kinase EnvZ and instead was phosphorylated by the non-cognate kinase CpxA or RstB. Each kinase was autophosphorylated, purified away from unincorporated nucleotide, and tested for phosphotransfer. In our reaction conditions at a 1 minute time point, EnvZ phosphotransfers exclusively to OmpR, whereas CpxA and RstB phosphotransfer exclusively to CpxR and RstA, respectively (Figure 2).

We first substituted residues in OmpR at the positions within alpha helix-1 identified by mutual information analysis with the corresponding residues from CpxR and RstA to create OmpR(MI-CpxR) and OmpR(MI-RstA); in each case three amino acid substitutions were made in OmpR. The mutant OmpR(MI-RstA) was not phosphorylated to a significant extent by RstB and was still a robust target of EnvZ (Figure 2A). The mutant OmpR(MI-CpxR) showed diminished phosphotransfer from EnvZ and was now phosphorylated by CpxA, although less efficiently than wild type CpxR (Figure 2B). The residues in alpha helix-1 are thus important for phosphotransfer specificity, but other residues must contribute.

We hypothesized that residues within the β5-α5 loop may also affect specificity of the regulator. One of these residues covaried strongly with residues in the histidine kinase (Figure 1) and other loop residues covaried at a slightly lower score threshold of 2.8. We thus swapped the residues in the OmpR loop with those from CpxR and RstA to create OmpR(MI+loop-RstA) and OmpR(MI+loop-CpxR), respectively, and examined phosphotransfer to each of these constructs; the former required three amino acid substitutions and the latter just one. Both constructs exhibited a nearly complete switch in phosphotransfer specificity. EnvZ was unable to phosphotransfer to either OmpR(MI+loop-RstA) or OmpR(MI+loop-CpxR), whereas phosphotransfer from RstB or CpxA to the respective rewired OmpR mutants was efficient and at near wild-type rates (Figure 2). Thus, the top coevolving residues appear sufficient, when mutated along with the β5-α5 loop, to rewire the phosphotransfer specificity of OmpR.

We note that the residues mutated to change the specificity of OmpR constitute a subset of the molecular interface formed by a cognate kinase and regulator (Figure 1D). For instance, the residues in the β4-α4 loop of the response regulator contact the histidine kinase, are in close proximity to the top coevolving residues, and coevolve with sites in the kinase at lower score thresholds (Figure S2), but mutating them was not required to change phosphotransfer specificity (Figure 2). We conclude that the strongest coevolving residues are necessary and sufficient to change the phosphotransfer partnering specificity of OmpR. Other residues may fine-tune the interaction, but do not make major contributions.

### Alanine-scanning mutagenesis and the role of individual residues

**Figure 3:**
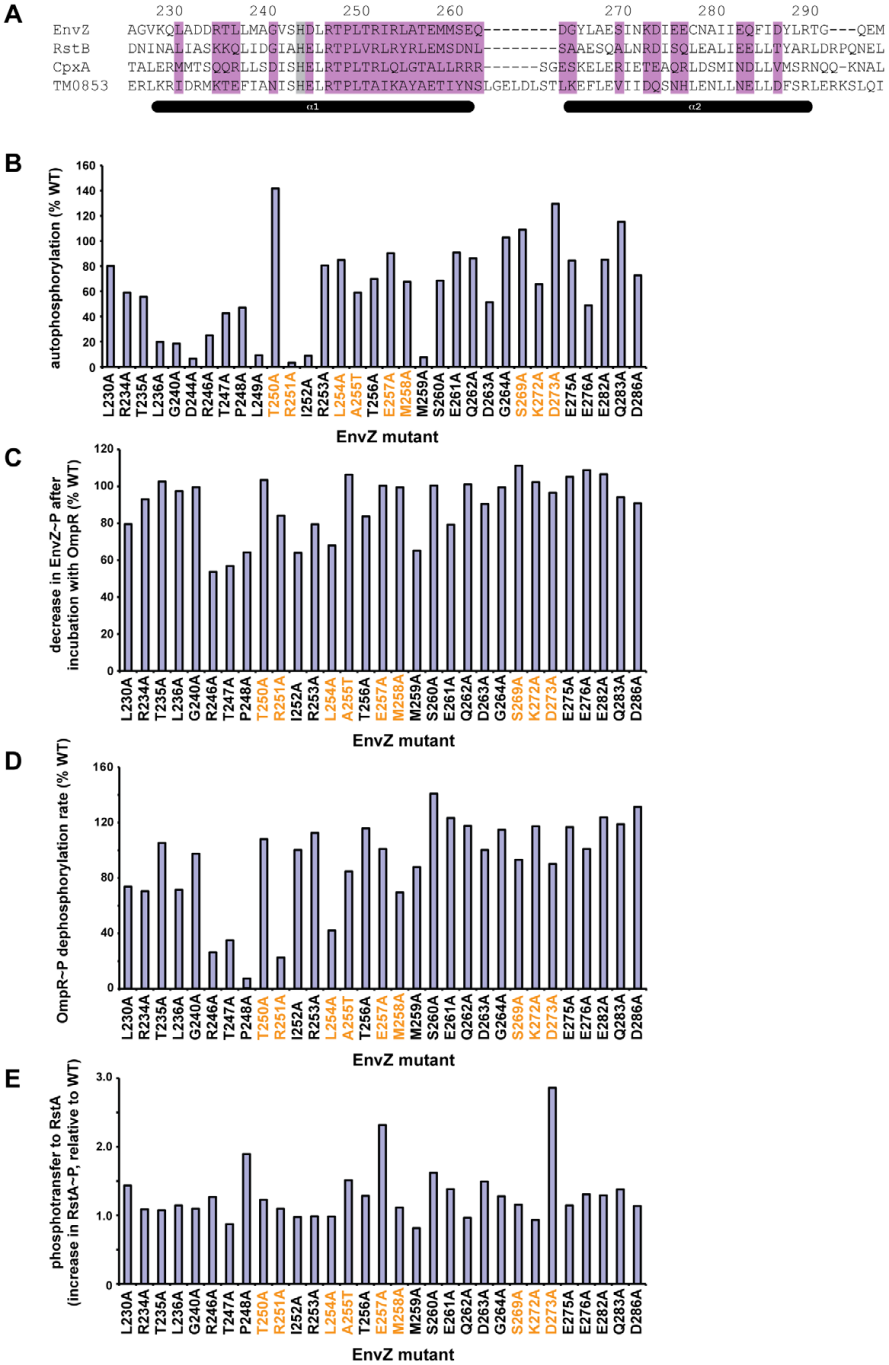
Alanine-scanning mutagenesis of EnvZ. (A) Sequence of the DHp domain of EnvZ showing the residues substituted with alanine in purple. The conserved histidine phosphorylation site is shaded in grey. Numbering and secondary structure elements indicated as in Figure 1C. (B) Autophosphorylation levels of each EnvZ alanine mutant after a 1 minute incubation, expressed as a percentage of that measured for wild-type EnvZ. For gel images, see Figure S3A. (C) Decrease in EnvZ∼P band after incubation with OmpR. Each value was expressed as a percentage of the decrease measured for wild-type EnvZ. Mutants that do not show a decrease in EnvZ∼P could be defective either in phosphotransfer or in dephosphorylation of OmpR∼P (see text for details). (D) Phosphatase activity of EnvZ alanine mutants. Each alanine mutant was tested for dephosphorylation of OmpR∼P and the rate expressed as a percentage of that measured for wild-type EnvZ. (E) Phosphotransfer from EnvZ alanine mutants to RstA. Phosphotransfer was assessed by measuring the increase in labeled RstA after a 10 second incubation. For each mutant, the increase in RstA was normalized to the autophosphorylation level for that kinase and then reported as a fold-change relative to the phosphotransfer for wild-type EnvZ to RstA. In panels B-E, the specificity residues are listed in orange, as in Figure 1C. For panels C and E, the mutant kinases were autophosphorylated for 60 minutes prior to assessing phosphotransfer. Mutants D244A and L249A did not autophosphorylate significantly enough to examine phosphotransfer. For gel images for panels C–D, see Figure S3B. For panel D, the mutant kinases were tested for dephosphorylation of OmpR∼P at 0.5, 1, and 2 minutes (Figure S4).

Our results indicate that kinase-substrate interaction specificity in two-component pathways is determined by a relatively small set of residues. But does each residue contribute equally to specificity or are there “hotspots” that contribute disproportionately? Do individual residues help bind the cognate substrate or help prevent interaction with non-cognate substrates? To address these questions, we performed alanine-scanning mutagenesis on the DHp domain of EnvZ. Surprisingly, despite being one of the best-characterized histidine kinases, EnvZ has never been explored through alanine-scanning mutagenesis. One study described a series of cysteine mutants [15], but the set of residues examined was limited and the interpretation of cysteine mutations can be ambiguous. We created a series of 33 EnvZ mutants to probe the role of most of the solvent-exposed residues in the DHp domain, generating alanine mutations for all residues except for A255, which was substituted with a threonine (Figure 3A).

We first examined the autophosphorylation activity of each EnvZ mutant (Figure 3B, Figure S3A). As expected, mutating the conserved phosphorylation site H243 (data not shown), or the highly conserved aspartate that follows, D244, completely abolished autophosphorylation. Other residues strongly affecting autophosphorylation flank H243, including L236, G240, R246, T247, P248, L249, R251, and I252. Many of these residues are highly conserved among all histidine kinases suggesting they are critical for catalyzing phosphoryl transfer from ATP to histidine. Alternatively, they may impact folding or stability of the kinase; however, these residues are mostly solvent-exposed and none of the mutants significantly affected purification of soluble protein (data not shown). Of the top coevolving residues (Figure 1), only R251A showed substantially lower autophosphorylation than wild type, suggesting that residues required for docking to a response regulator are distinct from those required for docking to the kinase's CA (catalytic ATP-binding) domain.

For each EnvZ mutant that was able to autophosphorylate to reasonably high levels after an extended incubation, we tested phosphotransfer to OmpR, CpxR, and RstA (Figure 3C–3E, Figure S3B). For an assessment of significance, see Figure S3C and Materials and Methods. For wild-type EnvZ, phosphotransfer to OmpR manifests as a decrease in the EnvZ∼P band and a weak or absent OmpR∼P band, resulting from high rates of phosphotransfer and subsequent dephosphorylation of OmpR∼P by EnvZ. Several alanine mutants did not show the same decrease in EnvZ∼P as the wild-type protein. However, for most of these mutants, such as R246A, T247A, and P248A, a more intense OmpR∼P band was also seen, suggesting that phosphotransfer had occurred but that the mutant could no longer dephosphorylate OmpR∼P. We confirmed the loss of phosphatase activity by measuring the dephosphorylation of purified OmpR∼P by each EnvZ mutant (Figure 3D, Figure S4). Only one mutant, I252A, showed a significant defect in phosphotransfer with no effect on phosphatase activity. Strikingly, mutating most of the coevolving specificity residues, including T250, R251, A255, E257, M258, S269, K272, and D273 had no major effect on phosphotransfer to OmpR. This finding suggests that there is no single “hot spot” and, instead, that specificity and molecular recognition are distributed over a number of residues. There may also be non-additive or synergistic effects between residues such that single point mutations do not significantly affect phosphotransfer in isolation, a possibility probed in more detail below.

Finally, we examined the EnvZ alanine mutants for phosphotransfer to the non-cognate regulators RstA and CpxR (Figure 3D, Figure S3B). For these reactions, in contrast to those shown in Figure 2, EnvZ constructs were autophosphorylated and tested for phosphotransfer without purifying them away from ATP. Under these conditions, EnvZ phosphotransfers weakly to RstA, permitting us to assess whether the alanine mutations affected this non-cognate interaction. Most mutants phosphorylated RstA at a level equivalent to or less than the wild type EnvZ. However, four mutants, P248A, A255T, E257A, and D273A, each showed increases in RstA phosphorylation; E257A also showed detectable phosphorylation of CpxR. Notably, three of the four residues were identified as specificity residues (Figure 1) in our coevolution analysis. The increase in cross-talk seen with these mutants suggests that these residues function, at least in part, as negative elements that prevent phosphotransfer to non-cognate substrates without significantly affecting transfer to the cognate substrate.

### Characterization of all intermediates along the mutational trajectories separating EnvZ and RstB

**Figure 4:**
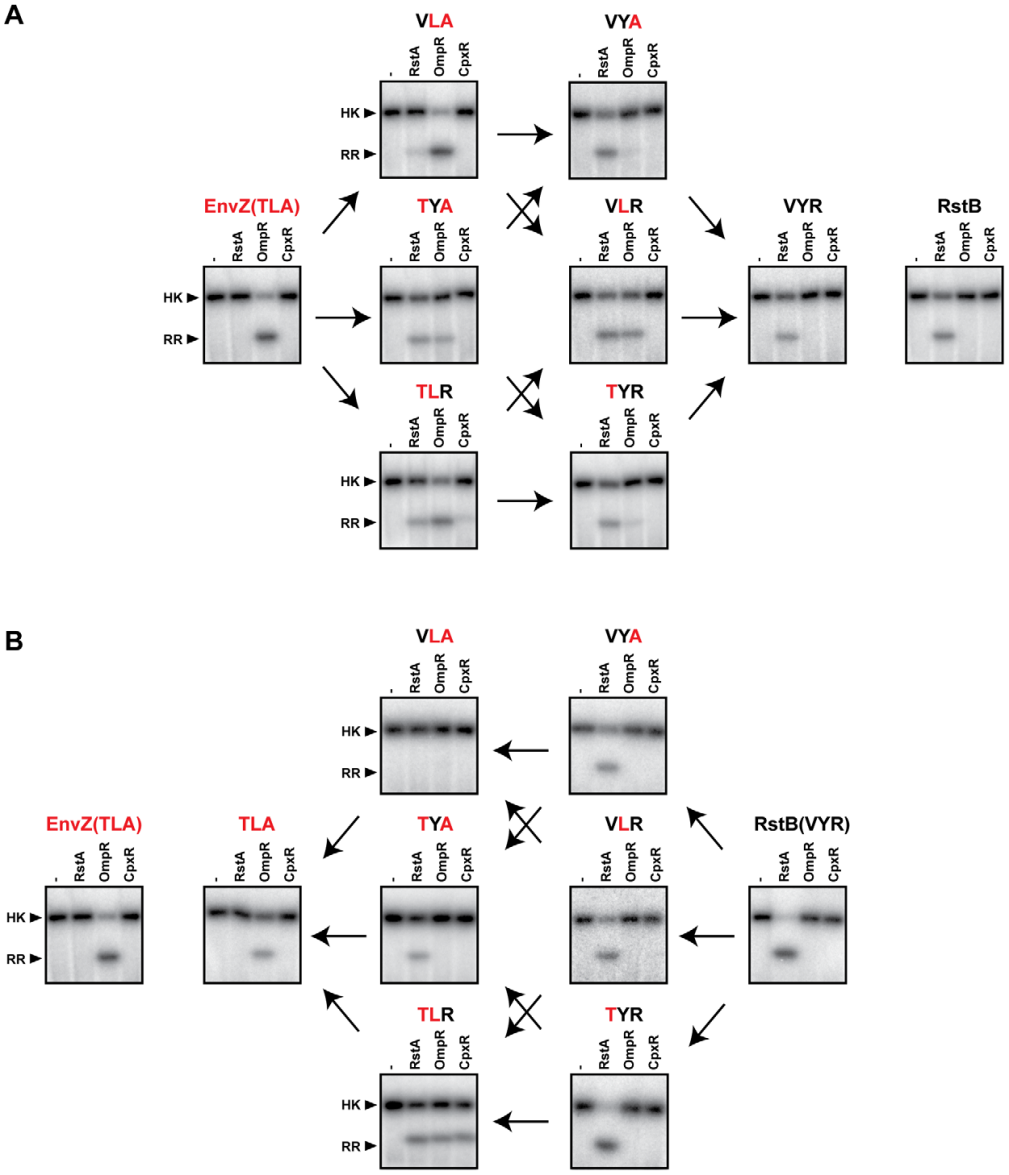
Converting the phosphotransfer specificity of EnvZ to match RstB and vice versa. (A) Converting the phosphotransfer specificity of EnvZ to that of RstB. Wild-type EnvZ and each single, double, and triple mutant on the trajectory from EnvZ to RstB were autophosphorylated and then incubated alone or with one of three response regulators, as indicated, for 10 seconds. Wild-type RstB (far right) is shown for comparison to EnvZ(VYR). (B) Converting the phosphotransfer specificity of RstB to that of EnvZ. Wild-type RstB and each single, double, and triple mutant on the trajectory from RstB to EnvZ was autophosphorylated and then incubated alone or with one of three response regulators, as indicated, for 60 seconds. Wild-type EnvZ (far left) is shown for comparison to RstB(TLA). Arrows connect profiles of mutants differing by a single amino acid substitution.

Although alanine-scanning provides some insight into specificity, an alanine substitution does not necessarily result in a simple loss of functionality, especially considering that EnvZ has a specificity residue that is already an alanine. In addition, as noted, there may be non-additive interdependencies between residues such that individual substitutions have minimal effect. We therefore sought to characterize the role of specificity-determining residues by examining the complete set of mutational intermediates between two histidine kinases with different specificities. For this analysis we focused on the paralogous systems EnvZ/OmpR and RstB/RstA, and term the approach trajectory-scanning. We constructed each possible specificity intermediate between EnvZ and RstB. This was feasible as the conversion of EnvZ phosphotransfer specificity to match that of RstB required only three substitutions, T250V, L254Y, and A255R [9]; the other major specificity residues identified by coevolution analysis are identical between EnvZ and RstB. In addition, we were able to rewire the specificity of RstB to match that of EnvZ by mutating the same three sites (Figure 4). The triple mutant RstB(V228T, Y232L, and R233A) no longer phosphorylated RstA and, instead, efficiently phosphorylated OmpR. These three residues thus play the dominant roles in dictating the specificity of both EnvZ and RstB. Other residues may make minor contributions.

We constructed each possible single and double mutant intermediate between EnvZ and RstB, in the context of each protein for a total of 12 mutants. To simplify nomenclature we have named mutants based on the protein mutated and the identity of the three specificity residues being considered. For example, wild-type EnvZ is EnvZ(TLA) and the single point mutant EnvZ(T250V) is EnvZ(VLA). Each mutant was tested for phosphotransfer to the regulators OmpR, RstA, and CpxR (Figure 4). Under the conditions used, the wild type EnvZ and RstB are specific for, and only phosphorylate, their cognate substrates, OmpR and RstA, respectively.

In the context of EnvZ, each single mutant continued to phosphorylate OmpR (Figure 4A). The single mutants EnvZ(TYA) and EnvZ(TLR) also showed weak phosphorylation of RstA. Of the double mutants, EnvZ(VYA) and EnvZ(TYR) both preferentially phosphorylated RstA, with the former not detectably phosphorylating OmpR and the latter only weakly phosphorylating OmpR. The other double mutant, EnvZ(VLR) appeared to have an approximately equal preference for phosphotransfer to RstA and OmpR. In the context of RstB, none of the three single mutants had a major effect on specificity and each continued to phosphotransfer only to RstA (Figure 4B). By contrast, the double mutants each behaved differently; the mutant RstB(TYA) phosphorylated only RstA, the mutant RstB(TLR) was promiscuous and phosphorylated RstA, OmpR, and CpxR, while the mutant RstB(VLA) did not phosphorylate any of the response regulators under these reaction conditions.

The systematic mapping of the mutational trajectories from EnvZ to RstB and *vice versa* led to several interesting observations (Figure 4). First, the behaviors of intermediates along individual trajectories are often quite different. The most dramatic example is the double mutants of RstB, with RstB(TLR) phosphorylating all three substrates examined, RstB(TYA) phosphorylating only RstA, and RstB(VLA) not phosphorylating any of the substrates. Second, we found that the individual specificity residues strongly influence each other. For example, the substitution V228T in the wild type RstB had very little effect on substrate preference, while the same substitution into RstB(VLA) converted a kinase that phosphorylated none of the regulators into a kinase that specifically phosphorylates OmpR (Figure 4B). The effect of the V228T substitution thus depends critically on the identity of other residues. As another example, the substitution Y230L in wild type RstA had little effect on specificity, but when introduced into RstA already harboring the V228T substitution produced a kinase that phosphorylated OmpR, RstA, and CpxR (Figure 4B). Similar observations were made for each of the other residues. Collectively, these data indicate that each specificity residue does not contribute independently or additively to the overall substrate specificity of a kinase. Rather, their contributions are frequently epistatic to one another and display context-dependence.

### A complete specificity map of the mutational trajectories separating EnvZ/OmpR and RstB/RstA

**Figure 5:**
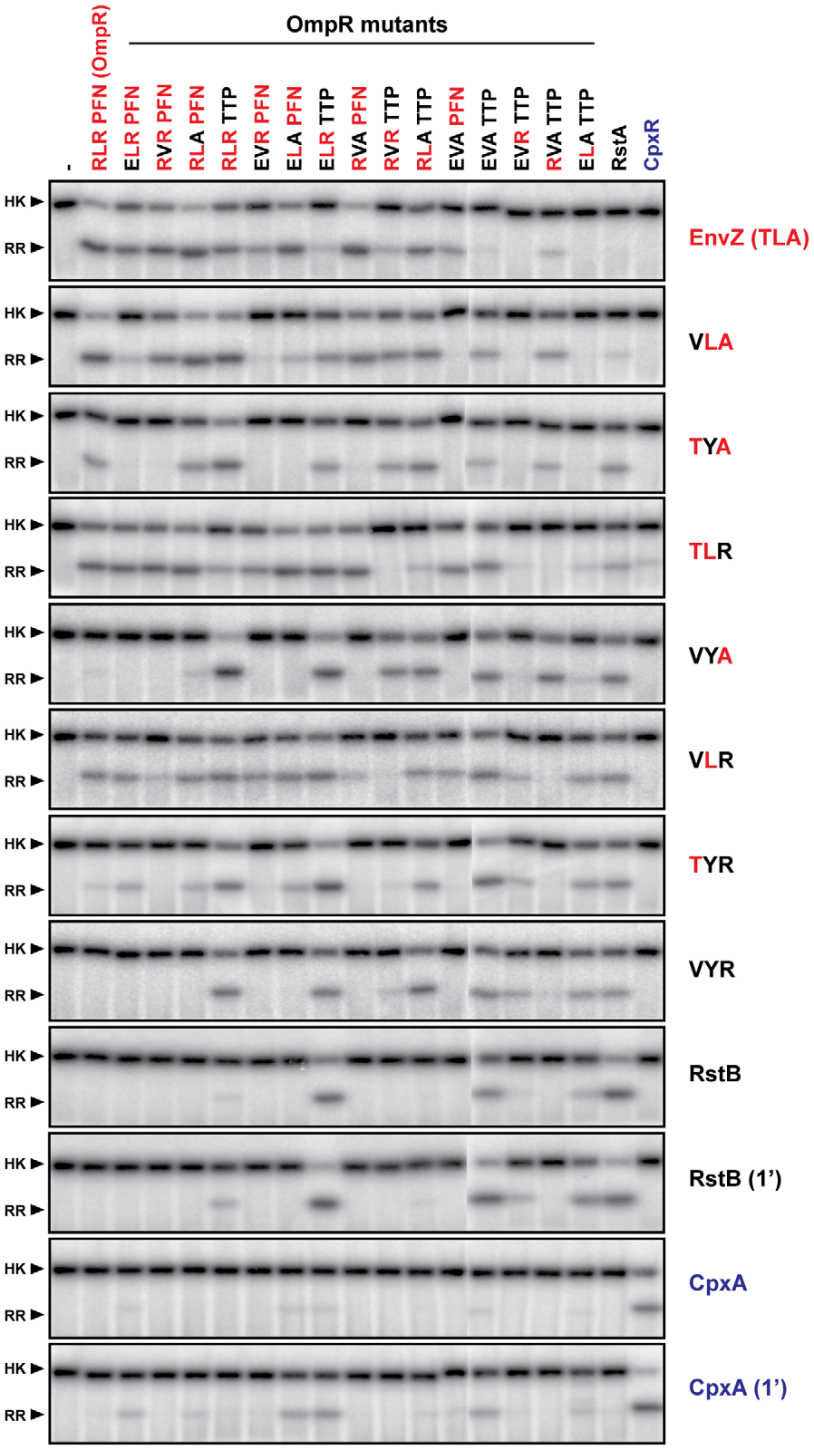
Complete trajectory-scanning mutagenesis of EnvZ and OmpR. Each histidine kinase, indicated on the far right, was autophosphorylated and tested for phosphotransfer to each of the response regulators listed across the top. Mutants of EnvZ are named according to the identity of the three specificity residues being examined; for instance, wild-type EnvZ is ‘TLA’ whereas the mutant T250V is ‘VLA’. Mutants of OmpR are named similarly. All phosphotransfer reactions were incubated for 10 seconds with the exception of RstB and CpxA, which were examined at both 10 seconds and 1 minute. Each kinase profile was composed of two separate gels that were run, exposed to phosphor screens, and scanned in parallel. The resulting two gel images were treated identically and then stitched together between OmpR(EVAPFN) and OmpR(EVATTP).

**Figure 6:**
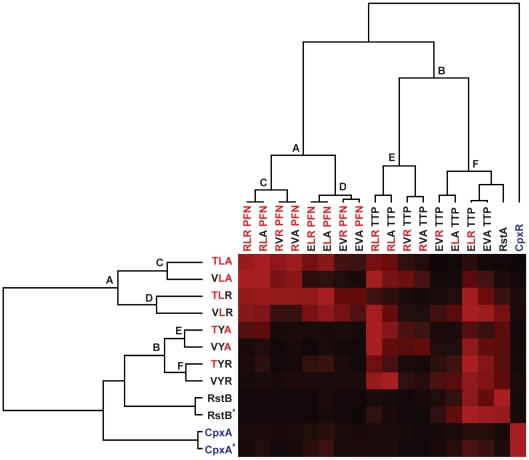
Hierarchical clustering of trajectory-scanning mutagenesis of EnvZ and OmpR. Phosphotransfer profiles for each EnvZ construct examined in Figure 5 were quantified. The intensity of each response regulator band within a given kinase profile was expressed as a percentage of the maximally phosphorylated response regulator in that profile. Profiles were then clustered in two-dimensions, with the resulting tree shown for the response regulators (top) and histidine kinases (left). For each tree, the major clusters of EnvZ and OmpR mutants are designated by letters. The 1 minute time point profiles for RstB and CpxA are indicated by ‘∧’.

The mutational trajectory scanning done for both EnvZ and RstB was extended to the response regulator OmpR. Converting OmpR to have the phosphotransfer specificity of RstA required 3 mutations in alpha helix-1 and 3 mutations in the β5-α5 loop (Figure 2A). We treated the loop as a single entity and made the 15 possible OmpR-RstA intermediates: 4 single, 6 double, 4 triple, and 1 quadruple mutant. We then examined phosphotransfer from each of the 7 EnvZ-RstB mutants (Figure 4A), as well as wild type EnvZ, RstB, and CpxA, to each of the 15 OmpR mutants and to wild-type OmpR, RstA, and CpxR, for a total of 180 pairwise combinations. The complete data are shown in Figure 5 and Figure 6. All phosphotransfer reactions were run for 10 seconds, except for RstB and CpxA, which were run for 10 seconds and for 1 minute. To evaluate phosphotransfer, we quantified the relative intensity of each response regulator band for a given histidine kinase, yielding a profile of phosphotransfer activity for each kinase. From the comprehensive profiles, several observations and trends emerged (Figure 5 and Figure 6).

First, the triple mutant EnvZ(VYR) robustly phosphorylated wild type RstA as well as the quadruple mutant of OmpR in which all major specificity residues have been mutated to match those found in RstA. EnvZ(VYR) no longer phosphorylated OmpR, consistent with a complete change in specificity. However, it still phosphorylated two other OmpR mutational intermediates that the wild type RstB kinase did not, at least at the time point examined. This comparison supports the notion that the three residues we mutated in EnvZ are the dominant determinants of partner specificity, but that other residues play minor, fine-tuning roles, particularly in preventing non-cognate interactions.

Second, the data demonstrated that EnvZ and OmpR can tolerate some mutations in the specificity residues of their partner and still retain the ability to readily phosphotransfer. Wild-type EnvZ phosphorylated each of the single mutants of OmpR and three of the six double mutants nearly as well as it phosphorylated wild-type OmpR; however, it did not significantly phosphorylate the triple mutants or the quadruple mutant. Wild-type OmpR was efficiently phosphorylated by each of the EnvZ single mutants and one of the double mutants, but not by the triple mutant.

**Figure 7:**
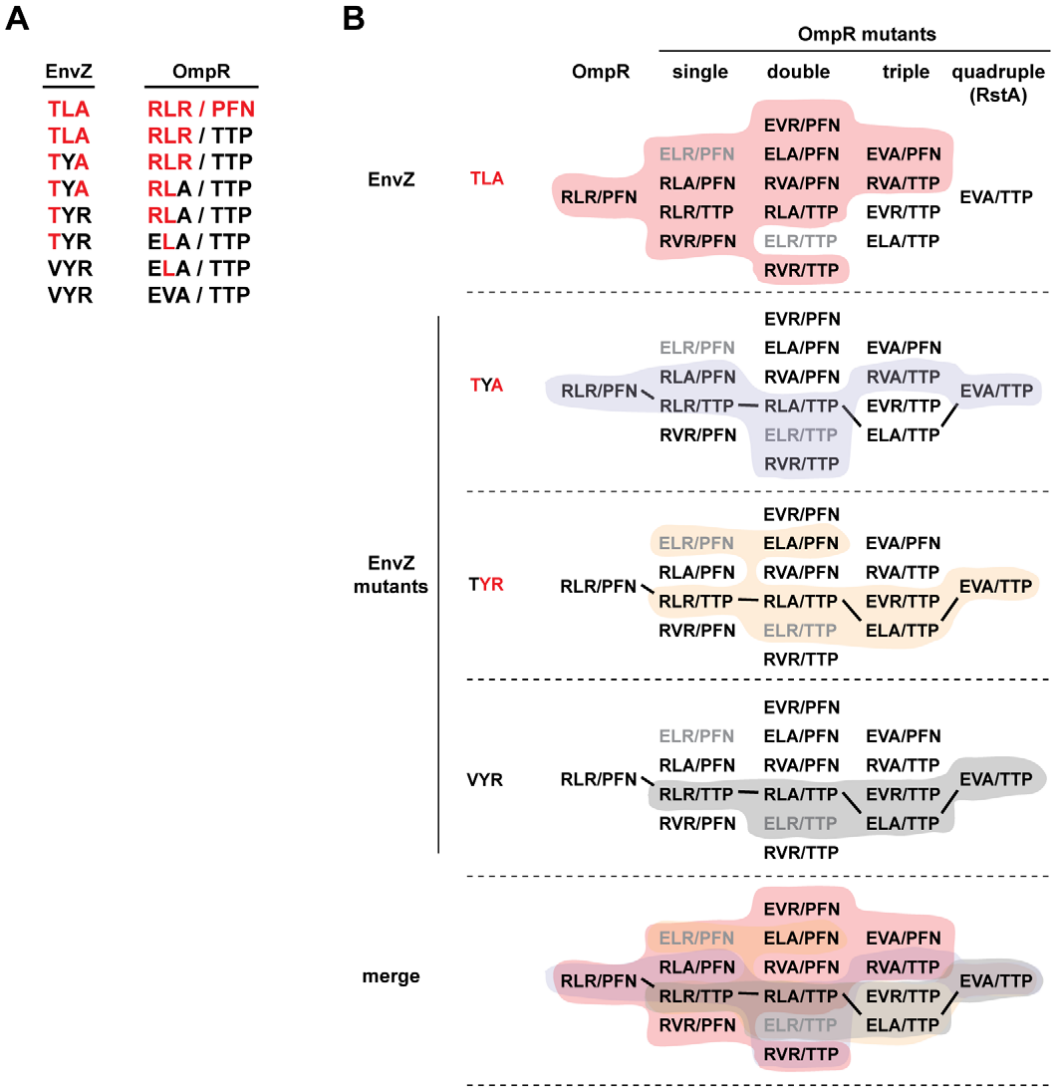
Mutational trajectories from EnvZ/OmpR to RstB/RstA. EnvZ and OmpR can be converted by a series of single mutations to harbor the specificity residues found in RstB and RstA, respectively, without disrupting phosphotransfer in intermediate stages. (A) A series of single mutations can convert the specificity of EnvZ to match that of RstB and OmpR to match RstA. Starting with the wild type specificity residues in red text at the top, each subsequent line introduces a single mutation (shown in black text) until both sets of specificity residues have been completely changed. As noted in the text, we treated the loop as a single mutation. As shown in panel B, each kinase-regulator pair listed is capable of phosphotransfer and does not include a regulator that is phosphorylated by CpxA. (B) The complete set of intermediates between wild type OmpR (RLR/PFN) and the quadruple mutant (EVA/TTP) are listed. For wild type EnvZ (TLA), the single mutant EnvZ(TYA), the double mutant EnvZ(TYR), and the triple mutant EnvZ(VYR), the set of OmpR mutants recognized by each kinase are shaded, with a merge of all four at the bottom. Mutants that are phosphorylated by CpxA are listed in grey text, all others in black text. Bold lines connect the mutant series shown in panel A.

Third, these profiles reveal mutational paths from the specificity of the EnvZ/OmpR pair to that of RstB/RstA in which phosphotransfer is maintained. In other words, there is an ordered series of single mutations that can be made in EnvZ and OmpR that convert them to the specificity of RstB and RstA, respectively, without disrupting their ability to phosphotransfer to one another along the way. For example, wild-type EnvZ phosphorylates OmpR and the single mutant OmpR(RLAPFN) to similar levels, and conversely the single mutant EnvZ(TLA) phosphorylates both OmpR and OmpR(RLAPFN). In Figure 7 we extend this example to show how EnvZ and OmpR could, in principle, change its specificity to that of the RstB/RstA system by a series of alternating mutations in the two molecules without ever severely disrupting their interaction. There are several such paths, although each path is not necessarily equivalent because CpxA phosphorylates some mutational intermediates of OmpR and some EnvZ mutants phosphorylate CpxR. For instance, EnvZ(TLR) phosphorylated CpxR, and OmpR(ELRPFN) was phosphorylated by CpxA (Figure 5, also see Figure 4). The avoidance of cross-talk may limit the possible evolutionary pathways between EnvZ/OmpR and RstA/RstB, or at least favor some relative to others (Figure 7).

We also quantified the phosphotransfer profiles for each EnvZ mutant and the wild type kinases (Figure 5) and performed hierarchical clustering in two dimensions, *i.e.* both the kinase and regulator dimensions (Figure 6). As expected, clustering the kinases places RstB close to the EnvZ(VYR) while CpxA is separated from EnvZ, the EnvZ mutants, and RstB. Similarly, clustering the regulators placed RstA close to the quadruple mutant OmpR(EVATTP) while CpxR formed a clear outgroup on its own.

The hierarchical clustering analysis provides insight into the relative importance of individual specificity residues. The profiles were clustered based on phosphorylation levels, but show a clear correspondence to sequence features. For instance, the two primary clusters of OmpR mutants (labeled A and B in Figure 6) differ in the identity of their β5-α5 loops; that is, each OmpR mutant in cluster A has the residues ‘PFN’ whereas each mutant in cluster B has the residues ‘TTP’. The branch lengths separating these clusters are long relative to the total length of the tree, indicating that the identity of the loop strongly splits the phosphotransfer profiles of the regulators. Within both cluster A and B, the next split in the tree correlates with the identity of position 1; that is, each OmpR mutant in cluster C (or cluster E) has an arginine at position 1 while each OmpR mutant in cluster D (or cluster F) has a glutamate at position 1. Again, the branch lengths are relatively long indicating a clear correlation between phosphotransfer behavior and sequence. The next split is based on identity at the second position, either a leucine or valine. The final split is based on the identity at the third position. In each case, this final split has extremely short branch lengths, reflecting the near identity of each profile pair that follows the split. In sum, the clustering analysis suggests a hierarchy to the contribution made by individual specificity residues within the regulators. The loop, which includes three residues, made the strongest contribution, followed by, in order, positions 1>2>3. A similar analysis was applied to the EnvZ mutants revealing that position 2 (Y or L) drives the initial clustering of EnvZ mutants, followed by position 3 (R or A), and finally position 1 (V or T).

## Discussion

### Determinants of specificity in paralogous protein families

Maintaining specificity and preventing unwanted cross-talk between highly similar proteins is a fundamental challenge for cells, and one that remains poorly understood. In many cases molecular recognition plays a critical role, but the ability to pinpoint the amino acids responsible and to determine the contributions of each residue to specificity has been elusive. Here, we tackled this problem in the context of bacterial two-component signal transduction systems where specificity is dictated by molecular recognition [6]. We note, however, that two-component signaling pathways are not insulated at all levels – for instance, distinct signaling pathways sometimes converge transcriptionally by regulating overlapping sets of genes [5]. However, the focus here is on the specificity of phosphotransfer for which there is little evidence of significant, physiologically-relevant cross-talk [5].

To identify the amino acids that enforce the specificity of phosphotransfer, we examined patterns of amino acid coevolution in cognate kinase-regulator pairs. However, computational approaches alone do not unequivocally establish which residues are critical for specificity or reveal how each contributes to substrate selection. We therefore focused on experimentally rewiring the specificity of the model two-component proteins, EnvZ and OmpR. Previously we reported that EnvZ could be rewired to exhibit the substrate specificity of RstB by mutating as few as three of the coevolving residues [9]. Here we extended these results by rewiring OmpR to partner specifically with the histidine kinase RstB instead of EnvZ.

The residues mutated to rewire the partnering specificity of EnvZ and OmpR are predicted to be in close physical proximity during phosphotransfer. While no structure of EnvZ bound to OmpR exists, a co-crystal structure of a histidine kinase from *Thermotoga maritima* in complex with its cognate response regulator was recently solved [13] and can be used to infer physically proximal residues for EnvZ and OmpR. However, the spatial proximity of residues does not reveal how they govern specificity and whether individual residues promote the binding of a cognate protein or prevent interactions with non-cognate proteins. Moreover, the relative contribution made by each residue is difficult to discern from structural or spatial considerations alone.

To better dissect the role played by individual residues, we used alanine-scanning mutagenesis of EnvZ. However, of the nine major specificity residues in EnvZ (Figure 1), only one disrupted phosphotransfer to OmpR when mutated to alanine. These data suggest that no major hot spot exists for the EnvZ-OmpR interaction and that specificity is distributed across the interface. However, single alanine mutants do not always reveal the role of a particular residue. For example, EnvZ(L254A) showed very little change in substrate specificity, whereas EnvZ(L254Y) (Figure 4A) showed a significant level of cross-talk to RstA. Alanine-scanning mutagenesis also ignores any potential interdependencies that may exist between residues. Such relationships and non-additive effects on specificity were revealed in our comprehensive characterization of the mutational intermediates separating EnvZ and RstB. In several cases, the effect of a given substitution on phosphotransfer specificity depended significantly on what other substitutions had already been made; for example the mutation A255R in EnvZ had very little effect in the context of EnvZ(VYA) but led to significant promiscuity in the context of EnvZ(TLA). These sorts of contextual and epistatic effects have been seen in other studies of molecular interaction specificity including corticosteroid receptor-ligand interaction [16] and transcription factor-DNA binding [17]. In principle, the context dependence of amino acids could lead to ‘negative’ epistasis in which one mutation on its own is detrimental until a second mutation is introduced. For example, the protein β-lactamase has evolved resistance to cefotaxime by accumulating five different mutations [18]. While each mutation contributes to resistance, certain mutations actually decrease resistance unless, or until, one of the other mutations also occurs. We did not see any obvious case of negative epistasis when converting EnvZ to RstB or converting OmpR to RstA, as each mutation either increased interaction with the target molecule or had no effect. However, negative epistasis could exist when converting the specificity of other two-component signaling proteins.

### Evolutionary implications

Our trajectory-scanning analysis provides a glimpse into the possible evolutionary history of two-component signaling proteins. The EnvZ/OmpR and RstB/RstA systems are relatively closely related and likely evolved by duplication of a common progenitor followed by sequence divergence, including at specificity sites. Mutations in specificity residues following duplication presumably required corresponding changes in their cognate regulators in order to maintain operation of each pathway as they diverged from one another to avoid pathway cross-talk. Our results demonstrate that an ordered series of mutations could occur in EnvZ and OmpR such that the two proteins would maintain significant levels of phosphotransfer while transiting through sequence space to the specificity residues of RstB/RstA (Figure 7), or *vice versa*. In addition, this series of mutations can occur without ever entering the sequence space occupied by another closely related (in sequence) pair, CpxA/CpxR thereby preventing cross-talk. Interestingly though, not all mutational trajectories have these characteristics of maintaining phosphotransfer and avoiding cross-talk, raising the possibility that sequence evolution following duplication is constrained or that natural selection may have favored certain trajectories over others. Analysis of other proteins, including β-lactamase, lambdoid phage integrases, hormone receptors, and the metabolic enzyme isopropylmalate dehydrogenase [18]-[21], have led to similar suggestions about the constraints on protein evolution.

Our trajectory scanning approach is related to other systematic studies of protein-protein interaction specificity, including homolog-scanning [22] and site-saturation mutagenesis [23]. In many cases, however, such approaches involve single substitutions rather than an exploration of the entire mutational landscape separating two different proteins. Because the major specificity-determining residues of two-component signaling proteins have been previously mapped and are relatively limited in number, we were able to systematically generate all intermediates between EnvZ/OmpR and RstB/RstA. We note, however, that for the three major specificity residues in EnvZ, T250, L254, and A255, conversion to the corresponding residue in RstB requires two nucleotide substitutions. There are thus a great number of additional mutational intermediates that will be important to characterize in the future when considering the evolutionary history of EnvZ and RstB.

Intriguingly, our clustering analysis of the trajectory-scanning data also reveals an underlying hierarchy of the specificity-determining residues in EnvZ and OmpR. The clusters mapped based on phosphotransfer relationships were strongly correlated with the sequence of specificity residues. For example, the first branch point in the histidine kinase clusters separated those with a leucine at position 254 in EnvZ from those with a tyrosine at that position. These observations demonstrate that different residues contribute unequally to specificity. So although our alanine-scanning mutagenesis did not reveal any major hot spots and suggested that specificity is distributed, the trajectory-scanning study indicates that certain residues play more important roles than others. It will be interesting to see whether the hierarchies revealed here have influenced or constrained evolutionary trajectories of two-component signaling proteins, and if the relative importance of positions is similar in other two-component pairs.

### Rational rewiring of two-component signaling pathways

The rational rewiring of two-component signaling proteins represents a stringent test of how well specificity is understood. Additionally, it opens the door to improved construction of synthetic signaling pathways in bacteria. Here, we used analyses of amino acid coevolution to guide the rational rewiring of the response regulator OmpR, a prototypical DNA-binding response regulator. With only a handful of mutations, the phosphotransfer specificity of OmpR was rewired to match that of RstA or CpxR. A recent study of *Rhodobacter* used structural data to guide the rewiring of chemotaxis response regulators to partner with the non-cognate kinase CheA_3_ [24]. The residues mutated in that study were in alpha helix 1 of the response regulator and most were identified here as coevolving residues. A genetic screen for altered partnering specificity of the regulator PhoB also identified residues in alpha helix 1 [25]. The successful rewiring of CheY and PhoB along with EnvZ and OmpR suggests that two-component proteins will be generally amenable to synthetic biology. However, it is not yet clear whether any histidine kinase (or response regulator) can be reprogrammed to behave like any other histidine kinase (or response regulator). For example, response regulators have been categorized into eight subfamilies, with the majority falling into just three [26]. OmpR, RstA, and CpxR all fall within one subfamily perhaps facilitating the interconversion of their specificities. Another important challenge for the future is to create novel kinase-regulator pairs with specificity residues that are orthogonal to those used in naturally occurring pairs. The functional hierarchies and interdependencies identified here will be important guides in engineering new, specific interactions. Similarly, these functional relationships should help in designing better algorithms for predicting kinase-regulator pairs in genomes of interest.

### Final perspective

The life of a cell depends critically on the specificity of protein-protein interactions. Yet we still have a relatively primitive understanding of how such specificity is encoded within proteins and how a set of amino acids can allow binding of a cognate partner while excluding all other non-cognate partners. Two-component signal transduction systems represent an ideal model for addressing these fundamental issues as specificity is determined predominantly by a small set of residues. The consequent reduction in scope and scale enabled the systematic and comprehensive analyses presented here. More generally, the approaches used, including analyses of amino acid coevolution and trajectory-scanning mutagenesis, will be widely applicable to the study of specificity and molecular recognition in many other protein-protein interactions.

## Materials and Methods

### Sequence analysis

The software HMMER (http://hmmer.org) was used, with an E-value cutoff of 0.01, to identify and align histidine kinase and response regulator sequences from fully sequenced bacterial genomes in GenBank. For histidine kinases, the models HisKA, HisKA_2, HisKA_3, and HWE_HK from the PFAM database were used. For response regulators, the model Response_reg was used. Histidine kinases and response regulators with GenBank genome identifier numbers differing by one, indicating adjacent genes, were identified, concatenated, and treated as cognate pairs. Sequences were filtered to ensure that no two sequences were more than 90% identical. The final set contained 4375 concatenated pairs of histidine kinase and response regulators. Columns in the multiple sequence alignment (MSA) containing greater than 10% gaps were eliminated.

Mutual information (MI) between columns was measured as described previously [9]. MI scores were adjusted to account for differences in the average MI of each column. For columns i and j in a multiple sequence alignment, we defined MI(i,j)_adj_ = MI(i,j)_raw_/(MI(i)_avg_+MI(j)_avg_)/2 where MI(i)_avg_ and MI(j)_avg_ are the average MI scores for column i and j paired with every other column in the alignment.

### Clustering

Phosphorylation profiles in Figure 6 were constructed by quantifying response regulator bands in each profile (Figure 5) using ImageQuant (GE Healthcare) and then normalizing such that each regulator's value was represented as a percentage of the maximally phosphorylated regulator for a given kinase. Profiles were then subjected to hierarchical clustering in two dimensions, with response regulators clustered using uncentered correlation and histidine kinases using Euclidean distance. Profiles were clustered using Cluster 3.0 [27] and visualized using Java Treeview [28].

### Protein purification

All cloning and site-directed mutagenesis was done with Gateway pENTR vectors (Invitrogen) following procedures described previously [9]. Mutagenesis primers are listed in Table S1. Clones in pENTR vectors were mobilized into destination vectors for expression and purification using Gateway LR reactions according to the manufacturer's protocol (Invitrogen). Histidine kinases were moved into pDEST-His_6_-MBP and response regulators into pDEST-TRX-His_6_. Expression and purification was carried out exactly as described previously [6].

### Autophosphorylation and phosphotransfer reactions

For autophosphorylation analysis of alanine mutants, histidine kinases were at a final concentration of 5 µM in HKEDG buffer (10 mM HEPES-KOH pH 8.0, 50 mM KCl, 10% glycerol, 0.1 mM EDTA, 2 mM DTT) supplemented with 5 mM MgCl_2_, 500 µM ATP, and 0.5 µCi [γ^32^P]-ATP from a stock at ∼6000 C_i_/mmol (Perkin Elmer). Reactions were incubated at room temperature for 1 minute, stopped by the addition of 4X loading buffer (500 mM Tris-HCl pH 6.8, 8% SDS, 40% glycerol, 400 mM β-mercaptoethanol), and analyzed by SDS-PAGE and phosphorimaging.

For phosphotransfer analysis, histidine kinases were autophosphorylated as above, but were incubated for 60 minutes at 30°C. Phosphotransfer was assessed by incubating autophosphorylated kinases with response regulators, each at a final concentration of 2.5 µM, at room temperature for the indicated time (either 10 seconds or 1 minute). Reactions were stopped by the addition of loading buffer, and analyzed by SDS-PAGE and phosphorimaging. For the experiments in Figure 2, Figure 4, and Figure 5, autophosphorylated kinases were purified away from unincorporated nucleotides by diluting them 1∶10 in HKEDG and then washing eight times in Nanosep 30K Omega columns (Pall Life Sciences) to minimize the effect of any phosphatase activity. The final eluate was diluted back to the original volume and MgCl_2_ added to 5 mM before assessing phosphotransfer.

For alanine-scanning mutagenesis, to gauge reproducibility and assess significance in the changes observed, we repeated the phosphotransfer reactions for wild type EnvZ six times and a subset of the mutants three times. Standard deviations in each case were ∼5–10% of the mean.

## Supporting Information

Figure S1 Adjusted mutual information analysis of amino acid covariation in two-component signaling proteins. (A) Histograms summarizing the raw mutual information scores for columns 8 and 270 in the kinase-regulator multiple sequence alignment against all other columns in the alignment. The arrow indicates the location of the score for the column pair 8-270. (B) Same as panel A, but for positions 18 and 202 in the alignment. (C) Scatterplot of raw mutual information scores against adjusted mutual information scores, as described in the main text and in Materials and Methods. Dashed line indicates the score cutoff of 3.5 used in Figure 1.
(0.08 MB PDF)

Figure S2 Identification of coevolving amino acids in cognate pairs of histidine kinases and response regulators. Same as Figure 1, except at a score threshold of 3.0. (A) Residues in histidine kinases and response regulators that strongly coevolve (adjusted MI score >3.0) are listed with lines connecting covarying pairs. Residues are numbered according to their position in *E. coli* EnvZ and OmpR and colored as in panels B-D. (B-C) Residues in histidine kinases that coevolve with residues in response regulators are shown on a primary sequence alignment of HK853 from *T. maritima* and EnvZ, RstB, and CpxA from *E. coli*. Residues in response regulators that strongly coevolve with residues in histidine kinases are shown on a primary sequence alignment of RR468 from *T. maritima* and OmpR, RstA, and CpxR from *E. coli*. Residues highly conserved across all two-component signaling proteins are shaded in grey. Coevolving residues above and below the phosphorylation site in the kinase are shown in green and orange, respectively. These two sets of residues coevolve with residues in the response regulator shaded in yellow and red, respectively. Secondary structure elements, based on the co-crystal structure of HK853 and RR468 from *T. maritima* [13], are shown beneath the sequences. (D) Coevolving residues mapped onto the HK853-RR458 structure. Coevolving residues are shown by space-filling and colored as in panels A-C. The side chains of the conserved phosphorylatable histidines and aspartate are shown as magenta sticks.
(0.21 MB PDF)

Figure S3 Alanine-scanning mutagenesis of EnvZ. (A) Each EnvZ mutant was autophosphorylated for 1 minute before reactions were stopped by the addition of loading buffer. Kinases were then examined by SDS-PAGE and phosphorimaging using four separate protein gels that were handled identically. Scanned images were concatenated; vertical bars separate lanes from different gels. For quantification, see Figure 3B. (B) Each EnvZ mutant was autophosphorylated for 60 minutes and then examined for phosphotransfer to OmpR, RstA, and CpxR. Phosphotransfer was assessed by measuring the decrease in labeled EnvZ after a 10 second incubation with OmpR. For quantification, see Figure 3C, 3E. (C) Reproducibility of phosphotransfer assays. Wild-type EnvZ was examined for phosphotransfer to OmpR and RstA six times while mutants T247A, L254A, and E257A were examined three times. The graph shows the mean and the individual values in red.
(0.36 MB PDF)

Figure S4 Dephosphorylation of OmpR∼P by EnvZ alanine mutants. Phosphorylated OmpR was purified and incubated with each EnvZ mutant for 0.5, 1, and 2 minutes. For a quantification of rates relative to wild-type EnvZ, see Figure 3D.
(0.24 MB PDF)

Table S1 Primers.
(0.02 MB PDF)
