# Supplementary figures and images for: Systematic Dissection and Trajectory-Scanning Mutagenesis of the Molecular Interface That Ensures Specificity of Two-Component Signaling Pathways
Source: PLoS Genet. 2010 Nov 24;6(11):e1001220. doi: 10.1371/journal.pgen.1001220 (PMC2991266; doi:10.1371/journal.pgen.1001220)

Figure S1

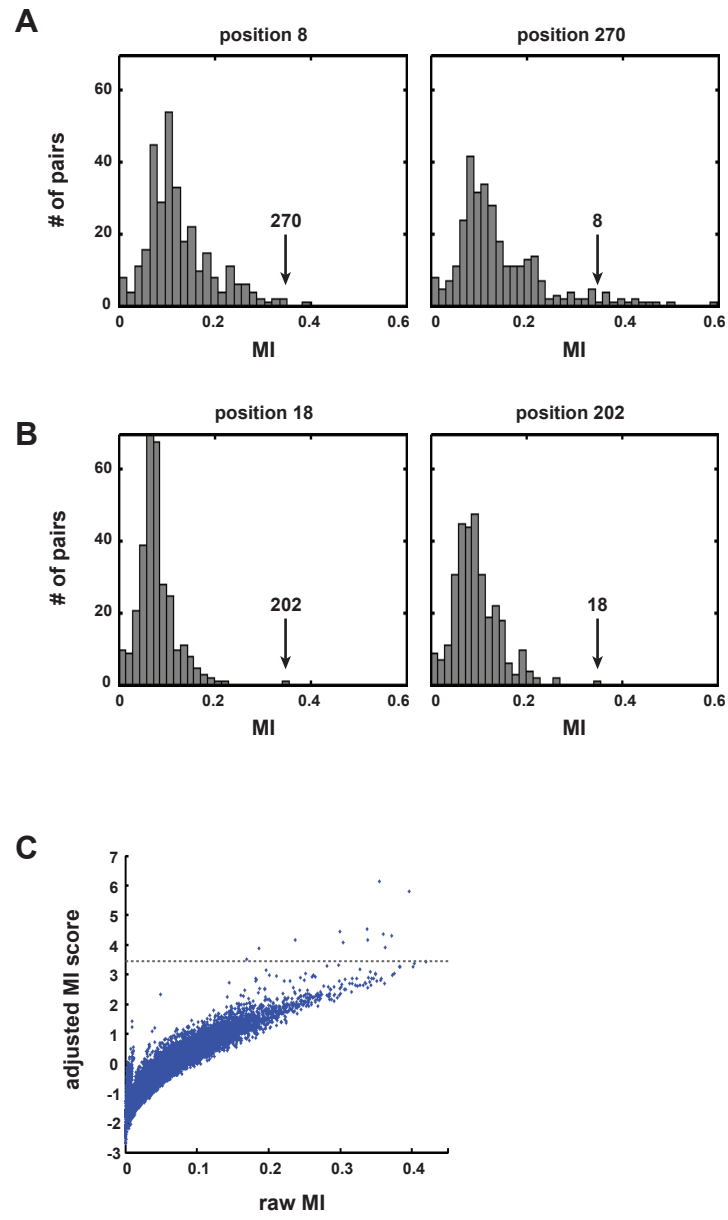

Supplement: Figure S1 — Adjusted mutual information analysis of amino acid covariation in two-component signaling proteins. (A) Histograms summarizing the raw mutual information scores for columns 8 and 270 in the kinase-regulator multiple sequence alignment against all other columns in the alignment. The arrow indicates the location of the score for the column pair 8-270. (B) Same as panel A, but for positions 18 and 202 in the alignment. (C) Scatterplot of raw mutual information scores against adjusted mutual information scores, as described in the main text and in Materials and Methods. Dashed line indicates the score cutoff of 3.5 used in Figure 1. (0.08 MB PDF) [file pgen.1001220.s001.pdf]

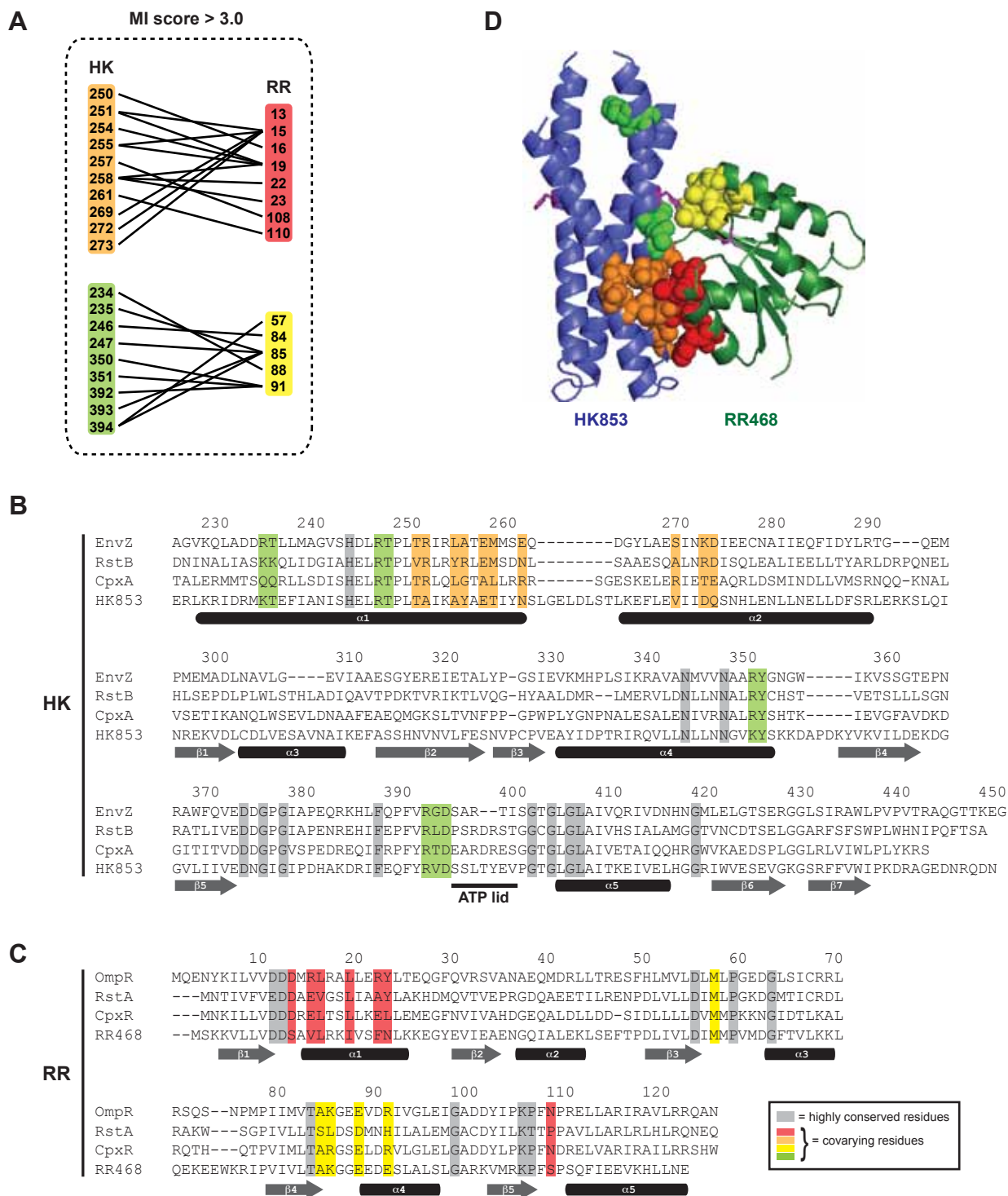

Supplement: Figure S2 — Identification of coevolving amino acids in cognate pairs of histidine kinases and response regulators. Same as Figure 1, except at a score threshold of 3.0. (A) Residues in histidine kinases and response regulators that strongly coevolve (adjusted MI score >3.0) are listed with lines connecting covarying pairs. Residues are numbered according to their position in E. coli EnvZ and OmpR and colored as in panels B-D. (B-C) Residues in histidine kinases that coevolve with residues in response regulators are shown on a primary sequence alignment of HK853 from T. maritima and EnvZ, RstB, and CpxA from E. coli. Residues in response regulators that strongly coevolve with residues in histidine kinases are shown on a primary sequence alignment of RR468 from T. maritima and OmpR, RstA, and CpxR from E. coli. Residues highly conserved across all two-component signaling proteins are shaded in grey. Coevolving residues above and below the phosphorylation site in the kinase are shown in green and orange, respectively. These two sets of residues coevolve with residues in the response regulator shaded in yellow and red, respectively. Secondary structure elements, based on the co-crystal structure of HK853 and RR468 from T. maritima [13], are shown beneath the sequences. (D) Coevolving residues mapped onto the HK853-RR458 structure. Coevolving residues are shown by space-filling and colored as in panels A-C. The side chains of the conserved phosphorylatable histidines and aspartate are shown as magenta sticks. (0.21 MB PDF) [file pgen.1001220.s002.pdf]

### Figure S3

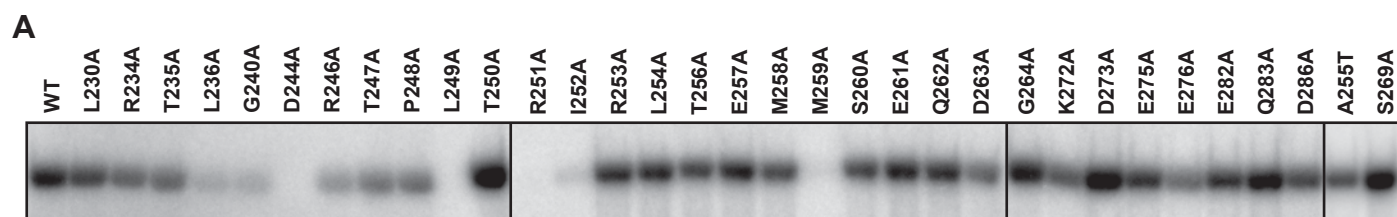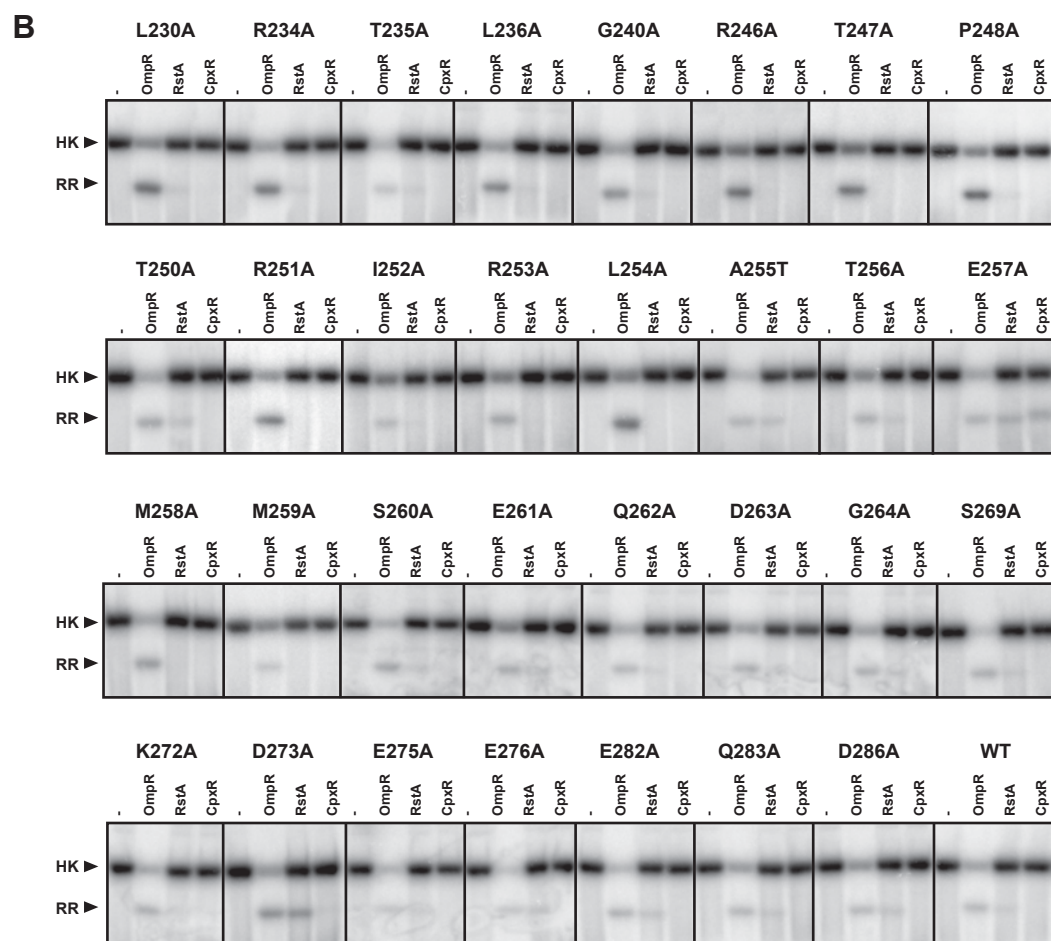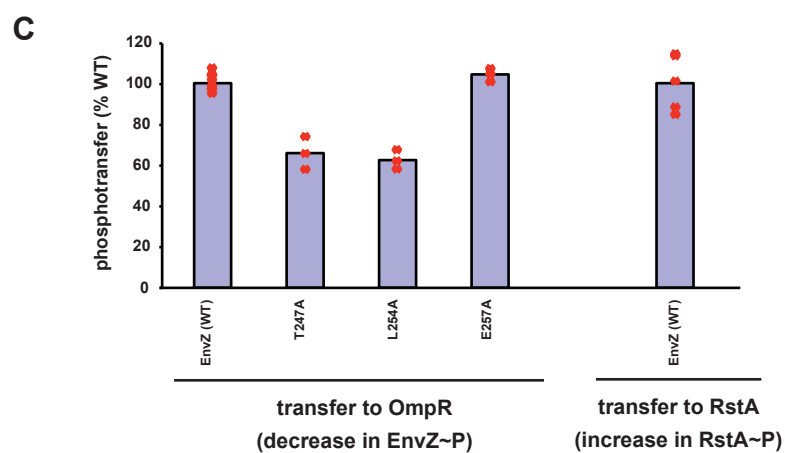

Supplement: Figure S3 — Alanine-scanning mutagenesis of EnvZ. (A) Each EnvZ mutant was autophosphorylated for 1 minute before reactions were stopped by the addition of loading buffer. Kinases were then examined by SDS-PAGE and phosphorimaging using four separate protein gels that were handled identically. Scanned images were concatenated; vertical bars separate lanes from different gels. For quantification, see Figure 3B. (B) Each EnvZ mutant was autophosphorylated for 60 minutes and then examined for phosphotransfer to OmpR, RstA, and CpxR. Phosphotransfer was assessed by measuring the decrease in labeled EnvZ after a 10 second incubation with OmpR. For quantification, see Figure 3C, 3E. (C) Reproducibility of phosphotransfer assays. Wild-type EnvZ was examined for phosphotransfer to OmpR and RstA six times while mutants T247A, L254A, and E257A were examined three times. The graph shows the mean and the individual values in red. (0.36 MB PDF) [file pgen.1001220.s003.pdf]

A

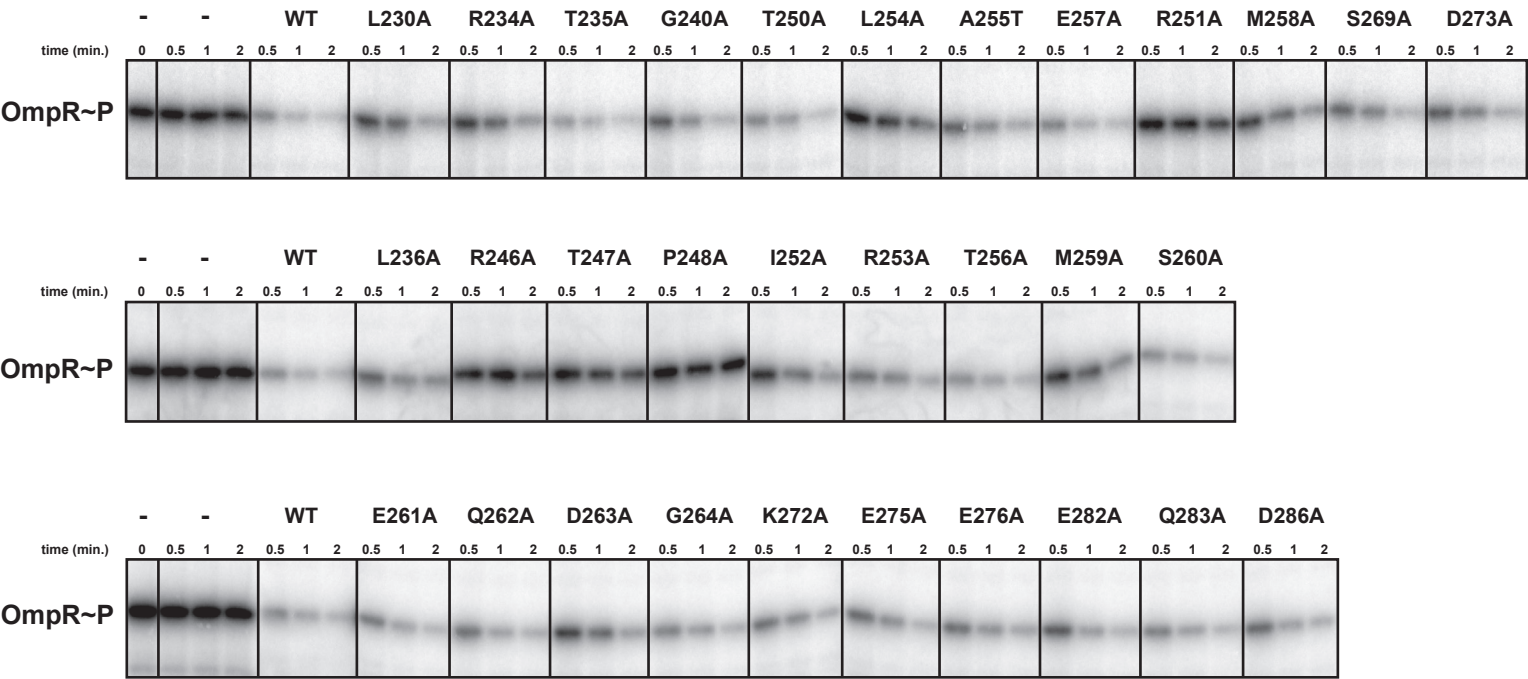

Supplement: Figure S4 — Dephosphorylation of OmpR∼P by EnvZ alanine mutants. Phosphorylated OmpR was purified and incubated with each EnvZ mutant for 0.5, 1, and 2 minutes. For a quantification of rates relative to wild-type EnvZ, see Figure 3D. (0.24 MB PDF) [file pgen.1001220.s004.pdf]
