# Supplementary material for: Systematic Dissection and Trajectory-Scanning Mutagenesis of the Molecular Interface That Ensures Specificity of Two-Component Signaling Pathways
Source: PLoS Genet. 2010 Nov 24;6(11):e1001220. doi: 10.1371/journal.pgen.1001220 (PMC2991266; doi:10.1371/journal.pgen.1001220)
Supplement: Table S1 — Primers. (0.02 MB PDF) [file pgen.1001220.s005.pdf]

Table S1 - Primers

Primer Name	Sequence*
OmpR(R15E)	GTCGATGACGACATGGAGCTGCGTGCGCTGCTG
OmpR(L16V)	GACGACATGCGCGTGCGTGCGCTGCTG
OmpR(R22A)	GCGCTGCTGGAAGCTTATCTCACCGAA
OmpR(R15E;L16V)	GATGACGACATGGAGGTGCGTGCGCTGCTG
OmpR(R22E;Y23L)	CGTGCGCTGCTGGAAGAACTGCTCACCGAACAAGGC
OmpR(P106T,F107T,N108P)	GACTACATTCCAAAAACGACGCCGCCGCGTGAACTGCTG
OmpR(P109D)	AAACCGTTTAACGACCGTGAACTGCTG
EnvZ(T250V)	ACGCCGCTGGTGCGTATTCGC
RstB(V229T)	CGAACACCGTTAACGCGCCTGCGTTAT
RstB(Y233L)	GTGCGCCTGCGTCCTCGACTGGAGATG
RstB(R234A)	CGCCTGCGTTATGCACTGGAGATGAGC
RstB(Y233L;R234A)	TTAGTGCGCCTGCGTCTTGCACTGGAGATGAGCGAT
RstB(Y233L)_onV229T	ACGCGCCTGCGTCTTCGACTGGAGATG
RstB(V229T;Y233L)_onR234A	CGAACACCGTTAACGCGCCTGCGTCTT
EnvZ(L230A)	GGTGTTAAGCAAGCGGCGGATGACCGC
EnvZ(R234A)	CTGGCGGATGACGCCACGCTGCTGATG
EnvZ(T235A)	TGGCGGATGACCGCGCGCTGCTGATGGCGGG
EnvZ(L236A)	CGGATGACCGCACGGCGCTGATGGCGGGGGT
EnvZ(G240A)	CGCTGCTGATGGCGGCGGTAAGTCACGACTT
EnvZ(D244A)	CGGGGGTAAGTCACGCGTTGCGCACGCCGCT
EnvZ(R246A)	TAAGTCACGACTTGGCGACGCCGCTGACGCG
EnvZ(T247A)	GTCACGACTTGCGCGCGCCGCTGACGCGTAT
EnvZ(P248A)	GACTTGCGCACGGCGCTGACGCGTATT
EnvZ(L249A)	TTGCGCACGCCGGCGACGCGTATTCGC
EnvZ(T250A)	TGCGCACGCCGCTGGCGCGTATTCGCCTGGC
EnvZ(R251A)	GCACGCCGCTGACGGCGATTCGCCTGGCGAC
EnvZ(I252A)	CCGCTGACGCGTGCTCGCCTGGCGACT
EnvZ(R253A)	CGCTGACGCGTATTGCGCTGGCGACTGAGAT
EnvZ(L254A)	ACGCGTATTCGCGCGGCGACTGAGATG
EnvZ(A255T)	CGTATTCGCCTGACGACTGAGATGATG
EnvZ(T256A)	ATTCGCCTGGCGGCTGAGATGATGAGC
EnvZ(E257A)	CGCCTGGCGACTGCGATGATGAGCGAG
EnvZ(M258A)	GCCTGGCGACTGAGGCGATGAGCGAGCAGGA
EnvZ(M259A)	TGGCGACTGAGATGGCGAGCGAGCAGGATGG
EnvZ(S260A)	CGACTGAGATGATGGCGGAGCAGGATGGCTA
EnvZ(E261A)	CTGAGATGATGAGCGCGCAGGATGGCTATCT
EnvZ(Q262A)	AGATGATGAGCGAGGCGGATGGCTATCTGGC
EnvZ(D263A)	TGATGAGCGAGCAGGCGGGCTATCTGGCAGA
EnvZ(G264A)	TGAGCGAGCAGGATGCGTATCTGGCAGAATC
EnvZ(S269A)	TATCTGGCAGAAGCGATCAATAAAGAT
EnvZ(K272A)	CAGAATCGATCAATGCGGATATCGAAGAGTG
EnvZ(D273A)	AATCGATCAATAAAGCGATCGAAGAGTGCAA
EnvZ(E275A)	TCAATAAAGATATCGCGGAGTGCAACGCCAT
EnvZ(E276A)	ATAAAGATATCGAAGCGTGCAACGCCATCAT
EnvZ(E282A)	GCAACGCCATCATTGCGCAGTTTATCGACTA
EnvZ(Q283A)	ACGCCATCATTGAGGCGTTTATCGACTACCT
EnvZ(D286A)	TTGAGCAGTTTATCGCGTACCTGCGCACCGG

*Site-directed mutagenesis was done using the primer listed as well as its reverse complement.
